# Novel pathological variants of NHP2 affect N-terminal domain flexibility, protein stability, H/ACA Ribonucleoprotein (RNP) complex formation and telomerase activity

**DOI:** 10.1093/hmg/ddad114

**Published:** 2023-07-13

**Authors:** Bartosz Maliński, Jacopo Vertemara, Elena Faustini, Claes Ladenvall, Anna Norberg, Yuming Zhang, Eleonore von Castelmur, Panagiotis Baliakas, Renata Tisi, Jörg Cammenga, Francisca Lottersberger

**Affiliations:** Department of Biomedical and Clinical Sciences, Faculty of Medicine and Health Sciences, Linköping University, Linköping 58185, Sweden; Dipartimento di Biotecnologie e Bioscienze, Università di Milano-Bicocca, Milan 20126, Italy; Department of Biomedical and Clinical Sciences, Faculty of Medicine and Health Sciences, Linköping University, Linköping 58185, Sweden; Department of Immunology, Genetics and Pathology, Uppsala University, Uppsala 90185, Sweden; Klinisk genetik, Norrlands Universitetssjukhus, Umeå 75185, Sweden; Department of Biomedical and Clinical Sciences, Faculty of Medicine and Health Sciences, Linköping University, Linköping 58185, Sweden; Department of Physics, Chemistry and Biology, Linköping University, Linköping 58183, Sweden; Department of Immunology, Genetics and Pathology, Uppsala University, Uppsala 90185, Sweden; Dipartimento di Biotecnologie e Bioscienze, Università di Milano-Bicocca, Milan 20126, Italy; Department of Biomedical and Clinical Sciences, Faculty of Medicine and Health Sciences, Linköping University, Linköping 58185, Sweden; Department of Laboratory Medicine, Lund University, Lund 22184, Sweden; Department of Biomedical and Clinical Sciences, Faculty of Medicine and Health Sciences, Linköping University, Linköping 58185, Sweden

## Abstract

Telomere biology disorders (TBDs) are characterized by short telomeres, premature aging, bone marrow failure and cancer predisposition. Germline mutations in *NHP2*, encoding for one component of the telomerase cofactor H/ACA RNA binding complex together with Dyskerin, NOP10 and GAR1, have been previously reported in rare cases of TBDs. Here, we report two novel *NHP2* variants (*NHP2-A39T* and *NHP2-T44M*) identified in a compound heterozygous patient affected by premature aging, bone marrow failure/myelodysplastic syndrome and gastric cancer. Although still able to support cell viability, both variants reduce the levels of hTR, the telomerase RNA component, and telomerase activity, expanding the panel of *NHP2* pathological variants. Furthermore, both variants fail to be incorporated in the H/ACA RNA binding complex when in competition with wild-type endogenous NHP2, and the lack of incorporation causes their drastic proteasomal degradation. By RoseTTAFold prediction followed by molecular dynamics simulations, we reveal a dramatic distortion of residues 33–41, which normally position on top of the NHP2 core, as the main defect of NHP2-A39T, and high flexibility and the misplacement of the N-terminal region (residues 1–24) in NHP2-T44M and, to a lower degree, in NHP2-A39T. Because deletion of amino acids 2–24 causes a reduction in NHP2 levels only in the presence of wild-type NHP2, while deletion of amino acids 2–38 completely disrupts NHP2 stability, we propose that the two variants are mis-incorporated into the H/ACA binding complex due to the altered dynamics of the first 23 amino acids and/or the distortion of the residues 25–41 loop.

## Introduction

Telomeres comprise thousands of TTAGGG duplexes bound by the six-protein shelterin complex and folded back in the t-loop structure (reviewed in 1). Due to the intrinsic inability of the DNA replication machinery to duplicate the DNA until the very end, telomeres progressively shorten during each cycle of cell division until they reach a critically short length, and cells enter into replicative senescence and/or apoptosis (reviewed in 2). The telomerase holoenzyme solves the end replication problem by adding TTAGGG repeats to the chromosome ends (3). The human telomerase consists of the reverse transcriptase hTert, the telomerase RNA component hTR, two H/ACA RNA binding complexes, the Cajal body protein TCAB/WRAP53 (reviewed in 2) and, as recently shown, histones H2A–H2B (4).

Germline mutations affecting telomere stability are associated with telomere biology disorders (TBDs) and are characterized by short telomeres and a spectrum of conditions, including premature aging, oral leukoplakia, skin pigmentation, nail dystrophy and bone marrow failure in dyskeratosis congenita (DC), intrauterine growth retardation and microcephaly in Høyeraal–Hreidarsson syndrome (HHS), and aplastic anemia (AA), idiopathic pulmonary fibrosis (IPF) and liver cirrhosis (reviewed in 5). Furthermore, TBDs patients show an increased predisposition to hematological malignancies and, more rarely, solid tumors (6–9). Due to the high heterogeneity of clinical features, TBDs are often underdiagnosed, and when diagnosed, the identification of the specific mutation/s involved fails in about 40% of patients, leading to suboptimal treatment (2,10,11).

The H/ACA RNA binding complex is formed by Dyskerin, NOP10, NHP2 and GAR1, and it binds H/ACA RNA hairpins co-transcriptionally (12–15). It is essential for the maturation of ribosomal RNA (rRNA) and small nuclear RNAs (snoRNAs) (16–21). Furthermore, two H/ACA RNA binding complexes are part of the telomerase holoenzyme, binding hTR and promoting its stability and maturation (22–25). NHP2, but not the other components of the complex, has also been associated with DNA damage response. However, the molecular mechanisms have not been elucidated yet (26–28).

Mutations in all the H/ACA RNA binding complex components, but GAR1, have been associated with TBDs (29–32). In particular, seven different pathogenic *NHP2* (OMIM #613987) variants have been identified in unrelated TBDs patients (29,31). Patients diagnosed with HHS or DC had biallelic *NHP2* mutations, whereas patients diagnosed with AA or interstitial lung disease were heterozygous. The resolved structures of the telomerase holoenzyme indicate that NHP2 interacts with NOP10 and hTR directly (4,24,25), and several TBD-associated mutations have been predicted to interfere directly with hTR binding. Consistently, reduced hTR stability and telomerase activity have been demonstrated with these variants (29,31).

In this study, we present two novel *NHP2* variants (*NHP2-A39T* and *NHP2-T44M*) identified in an adult patient diagnosed with DC clinical features, bone marrow failure/myelodysplastic syndrome (MDS) and gastric cancer, and we validate both of them as pathological for hTR expression and telomerase activity in two cancer cell lines. By molecular modeling and dynamics simulation, we predict that the first 41 amino acids of NHP2 are either too flexible or misplaced in both variants, potentially affecting their binding to hTR or NOP10. We then show that protein expression of both NHP2-A39T and NHP2-T44M is severely compromised in the presence of endogenous NHP2 due to reduced binding to NOP10 and Dyskerin and high proteasomal degradation, but it is restored when endogenous NHP2 is deleted. A similar phenotype is observed for an NHP2 mutant lacking the first 23 amino acids, whereas the deletion of all the first 37 amino acids completely compromises NHP2 protein levels. Therefore, we propose that all the NHP2 not incorporated in the H/ACA RNA binding complex is rapidly degraded by the proteasome and that the correct positioning of the first 24 amino acids of NHP2 plays a key role in the kinetics of such incorporation.

## Results

### Identification of two novel *NHP2* variants

**Figure 1:**
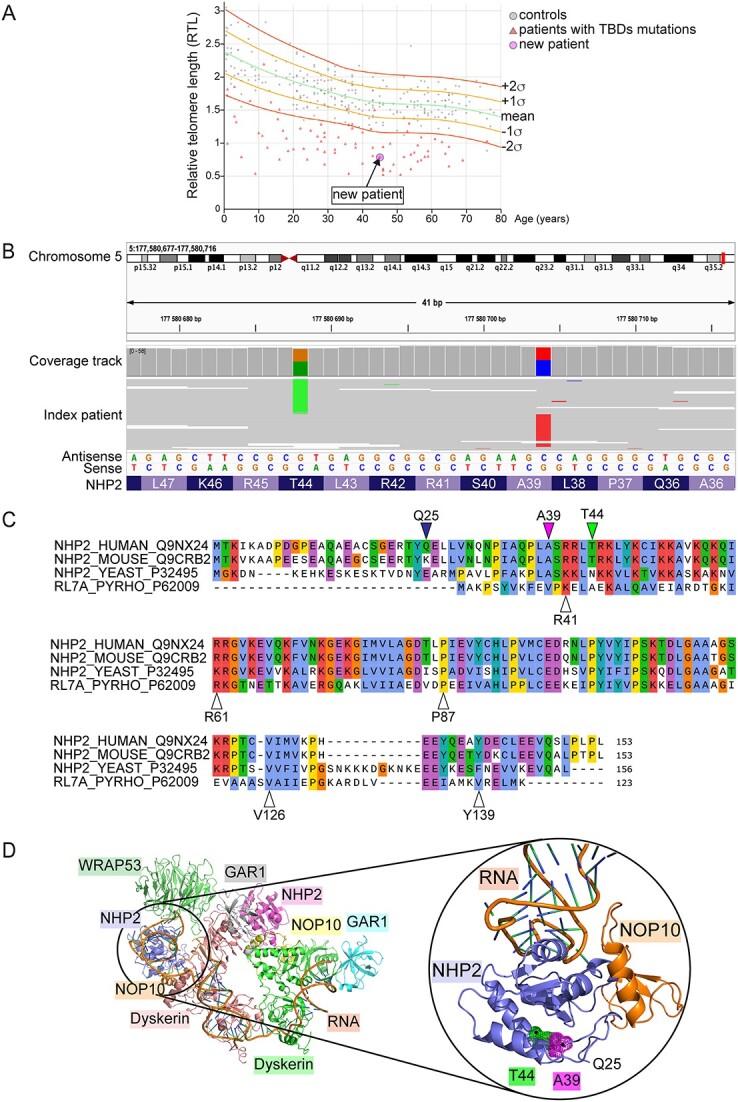
Identification of two new *NHP2* variants in a patient with short telomeres. (**A**) PCR for telomere length in the patient’s peripheral blood. The Relative telomere length (RTL) value of each sample is plotted against the individual’s age. Controls as gray circles, patients with TBD mutations as red triangles, arrow indicates the new index patient. (**B**) Visualization by Integrative Genomics Viewer of the *NHP2* variants identified in the index patient (antisense strand). c.131C > T (chr5:177580688) leading to pThr44Met (left) and c.115G > A (chr5:177580704) leading to p.A39T (right) are always on separate sequence reads and all reads have one of the variants present. (**C**) Clustal Omega Alignment of NHP2 isoforms from human, mouse and *Saccharomyces cerevisiae*, and RL7A from *Pyrococcus horikoshii*, with ClustalX coloring. Top arrows indicate the first amino acid resolved in the cryo-EM structure (Q25), A39 and T44. Bottom arrows indicate the residues previously found mutated in TBDs (29,31). (**D**) Cryo-EM structure (PDB ID: 7bgb) of H/ACA RNP lobe of human telomerase; blow-up shows NHP2 and NOP10 bound to hTR RNA. Highlighted are residues A39 and T44. The NHP2 structure starts from Q25.

The index patient with no family history of MDS was diagnosed at age 38 with gastric cancer and low-risk MDS, with normal karyotype (46,XY[20]). Cancer was successfully removed by surgery (Billroth II). After 7 years, his cytopenia (mainly anemia) aggravated. Further clinical investigation revealed nail dystrophies, gray hair, tooth loss and oral leukoplakia with a bone marrow cellularity between 30 and 40%, which decreased to 10–30% in the following 8 months, with normal karyotype but erythro- and megakaryopoiesis dysplasia, suggesting TBDs. Consistently, PCR showed short telomeres compared with same-age controls (Fig. 1A). Family’s samples were not available, and mutation analysis using Illumina TruSight Myeloid Sequencing panel Sanger sequencing of TERT and TERC coding exons failed to detect any pathogenic variant. The patient underwent allogeneic bone marrow transplantations but rejected the graft and, after a second transplantation, unfortunately died of Epstein–Barr virus infection.

Subsequent whole-genome sequencing performed on DNA extracted from the patient’s fibroblasts identified compound heterozygote missense variants in *NHP2* at c.115G > A (chr5:177580704) and c.131C > T (chr5:177580688) leading to p.Ala39Thr (hereafter indicated as *NHP2-A39T*) and p. Thr44Met (*NHP2-T44M*), respectively (Fig. 1B). The first variant is present in the European population with the very low frequency of 0.9 × 10^−4^ (33). However, it has never previously been associated with TBD. The second variant has never been reported before. No other variants of potential clinical significance were detected.

These two mutations are located in the N-terminal region of NHP2, in proximity to only one other previously identified *NHP2* mutation (R41H) (Fig. 1C) (29). Targeted genome BLASTp identified the A39 residue as conserved in eukaryotes but not in the archaea Ribosomal Protein L7 (RL7) paralogue, and T44 as conserved only in Tetrapods (Fig. 1C and Supplementary Material, Fig. S1). Although accordingly to the recently resolved cryogenic electron microscopy (cryo-EM) structure of the H/ACA Ribonucleoprotein (RNP) lobe of human telomerase (PDB ID: 7bgb (4)), neither residue A39 nor T44 seems to be directly involved in the interaction of NHP2 with NOP10 and/or hTR (Fig. 1D), both substitutions were predicted *in silico* to be highly deleterious for NHP2 protein function (SIFT tolerance score of 0.00 and PolyPhen confidence score of 0.999 for both).

### Both NHP2-A39T and NHP2-T44M affect hTR stability and telomerase activity

**Figure 2:**
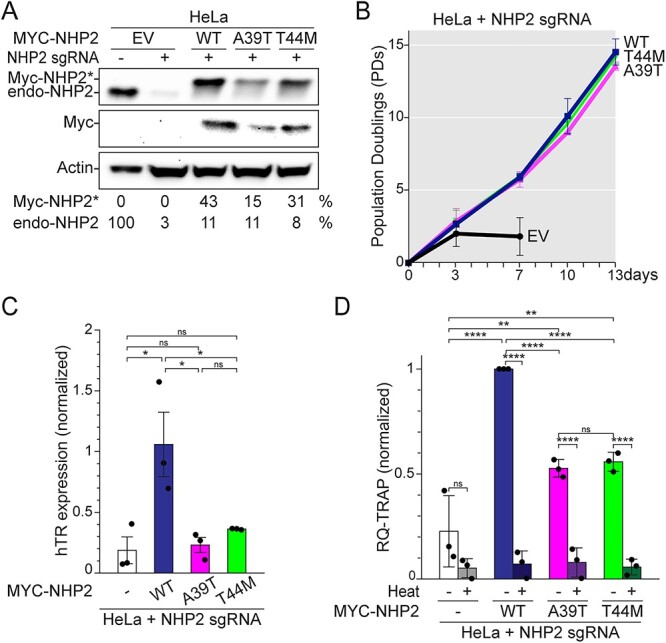
Reduced telomerase activity in cells expressing NHP2-A39T or NHP2-T44M. (**A**) Immunoblot for NHP2 and (**B**) survival curve for HeLa cells transduced with the empty vector (EV) or *MYC*-tagged *NHP2-WT*, *NHP2-A39T* or *NHP2-T44M*, before or after CRISPR/Cas9-mediated deletion of endogenous *NHP2*. The signal for Myc-NHP2 (Myc-NHP2^*^) or endogenous NHP2 (endo-NHP2) was normalized to the actin loading control and then to the value obtained for endo-NHP2 in untreated conditions. Day 0 is set 4 days after endo *NHP2* deletion. (**C**) qPCR for hTR and (**D**) Real-time quantitative telomeric repeat amplification protocol (RQ-TRAP) for *n* = 3 independent experiments, with average and SD. Values were normalized on Myc-NHP2-WT. Statistics: ordinary one-way ANOVA for multiple comparisons.

This investigation started after the patient was deceased, and no patient’s samples were available anymore. Therefore, we characterized the effect of *NHP2-A39T* and *NHP2-T44M* variants on telomerase activity in human cell lines. Because NHP2 is essential, *MYC*-tagged wild-type *NHP2* (*NHP2-WT*) or mutant *NHP2* (*NHP2-A39T* or *NHP2-T44M*) were expressed in telomerase-positive HeLa cancer cell line first, and then the bulk endogenous NHP2 was targeted with CRISPR/Cas9. The Myc-tag allowed to differentiate between exogenous Myc-NHP2 (NHP2^*^) and endogenous NHP2 (endo-NHP2) on the immunoblot (Fig. 2A). Consistent with previous reports (26,34,35), deletion of genomic *NHP2* by CRISPR/Cas9 caused a sudden arrest of growth (Fig. 2A and B). NHP2-WT complemented *NHP2* deletion, indicating that the Myc-tag did not affect NHP2’s essential functions (Fig. 2A and B). Although present at lower levels than NHP2-WT, both NHP2-A39T and NHP2-T44M could also support cell survival as wild-type NHP2 (Fig. 2A and B), indicating that their essential function was not impaired. Nevertheless, both caused lower hTR expression and a significant reduction in telomerase activity compared with NHP2-WT (Fig. 2C and D and Supplemental Material, Tables S1 and S2). Similar data were obtained with the cancer cell line HT1080 (Supplementary Material, Fig. S2A–D and Supplemental Material, Tables S3 and S4), validating both *NHP2-A39T* and *NHP2-T44M* as new TBD-causing *NHP2* variants.

### NHP2-A39T and NHP2-T44M do not affect cell survival after DNA damage

*NHP2* depletion has been previously shown to affect DNA damage response in cancer cells (26,27). However, neither of the two mutations caused a significant reduction in the survival of HeLa cells after treatment with the DNA-targeting drugs aphidicolin, neocarzinostatin or cisplatin when compared with NHP2-WT (Supplementary Material, Fig. S3A–C). Similar results were obtained after aphidicolin, γ-irradiation or cisplatin treatment in U-2OS cancer cell line (Supplementary Material, Fig. S3D–G), which survive through a telomerase-independent alternative lengthening of telomeres (ALT) mechanism (reviewed in 2,36), thus indicating that neither mutations compromise NHP2’s role in DNA damage response.

### A39T and T44M are predicted to affect the behavior of the NHP2 N-terminal tail

**Figure 3:**
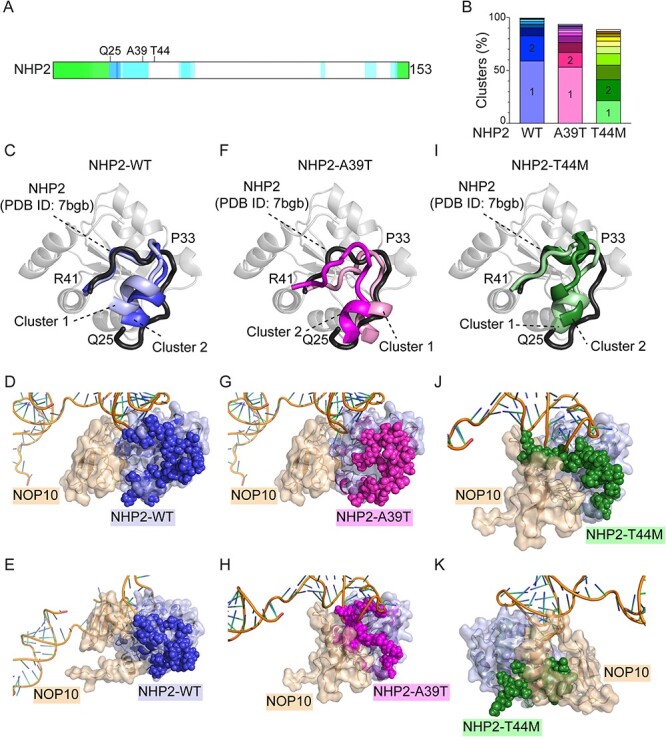
*In silico* analysis of NHP2^*^ conformations. (**A**) Schematics of the intrinsic disorder of NHP2 as generated by D^2^P^2^ database by consensus (**B**) Frequency distribution of the first 10 clusters identified by five simulation trajectories of 1 μs each for the wild-type NHP2, NHP2-A39T and NHP2-T44M. Superposition of the region between amino acids Q25 and R41 of the two most representative NHP2-WT clusters on NHP2 as resolved in (PDB ID: 7bgb) (**C**). Superposition of the centroid of NHP2-WT cluster 1 (**D**) and NHP2-WT cluster 2 (**E**) on NHP2 interacting with NOP10 and hTR as in (PDB ID: 7bgb). Superposition of two most representative clusters of NHP2-A39T (**F**–**H**) and NHP2-T44M (**I**–**K**) on NHP2 as resolved in (PDB ID: 7bgb) as described in (C–E). The structures are shown in different orientation to highlight the positioning of the N-terminal.

Because residues A39 nor T44 did not localize close to NOP10 or hTR in either of the two H/ACA heterodimers resolved in the cryo-EM structure of the telomerase (Fig. 1D), and both NHP2-A39T and NHP2-T44M were expressed to lower levels than NHP2-WT, we decided to investigate the possible effect of A39T and T44M mutations on NHP2 structure *in silico.* First, we used RoseTTAFold (37) to build initial structural models of full-length human NHP2, including the first 24 N-terminal amino acids, which have not been resolved in any structures so far and are predicted to be highly disordered (Fig. 3A) (38). All five generated models identified a protrusion of the N-terminal (Supplementary Material, Fig. S4A). However, the confidence was very low, with an estimated error higher than 1.2 Å due to the lack of any reference structure. Therefore, and considering the disordered nature of this region, we performed molecular dynamics (MD) simulations. To reach the wider exploration of the conformational space, five replicas, each one starting from one of the different RoseTTAFold models, were simulated for 1 μs each. The five independent resulting trajectories partially overlap, indicating that the N-terminal region of NHP2 could move from one starting point to the other during the simulation, adopting all the conformations proposed by the structure predictor (Supplementary Material, Movies S1–S5). Cluster analysis on the whole combined trajectories revealed that wild-type NHP2 explored two prominent configurations (Supplementary Material, Fig. S4B), accounting for up to the 82% of simulation time (Fig. 3B). From residue P33 both configurations were largely overlapping with the resolved cryo-EM structure of NHP2 in the telomerase holoenzyme (Fig. 3C and Supplementary Material, Fig. S4C), whereas the upstream residues could assume two relatively stable distinct conformations, neither of them interfering with the interface between NHP2 and NOP10 or hTR (Fig. 3C–E and Supplementary Material, Fig. S4B and C) and stabilized by a network of interactions between the N-terminal residues and the globular domain of NHP2 (Supplementary material, Fig. S5A).

**Figure 4:**
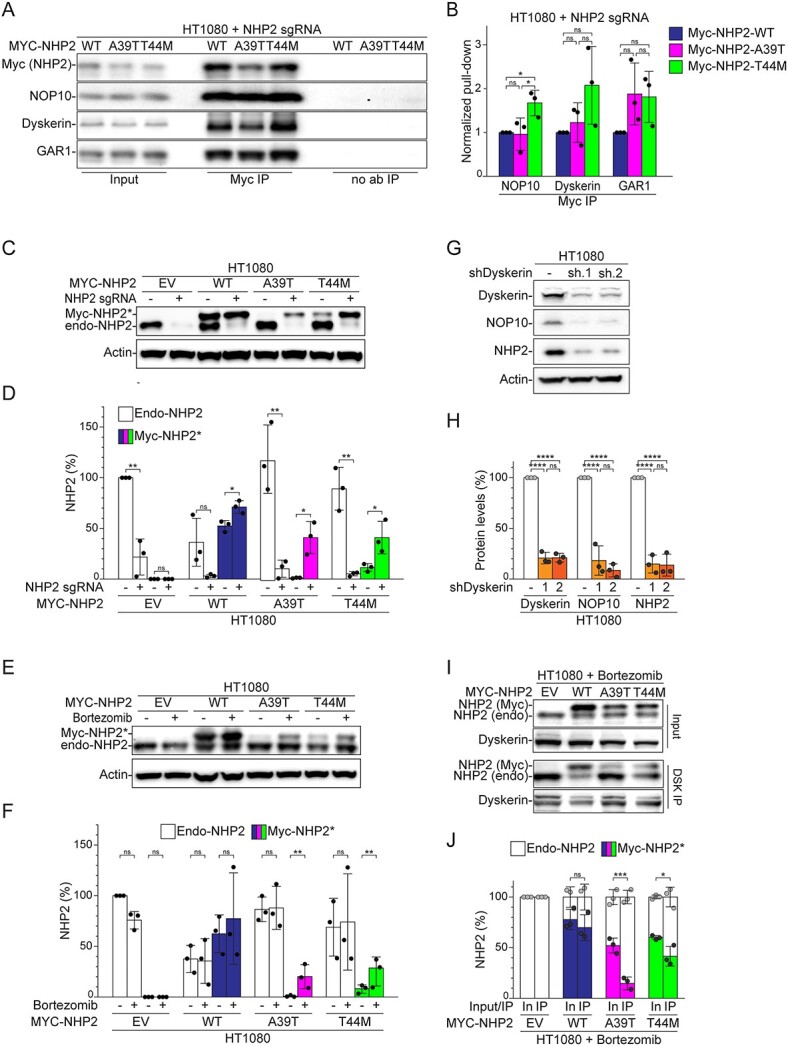
Reduced affinity and stability of NHP2-A39T and NHP2-T44M in the presence of endogenous NHP2. (**A**) Immunoblot for NHP2, NOP10, Dyskerin and GAR1 in HT1080 cells transduced with the indicated *MYC-NHP2* alleles after CRISPR/Cas9-mediated deletion of endo-*NHP2* before (input) and after pull-down with anti-Myc antibodies (Myc IP) or no antibodies (no ab IP). (**B**) Quantification of NOP10, Dyskerin and GAR1 pulled down with Myc antibodies as described in (A) for *n* = 3 independent experiments, with average and SD. NOP10, Dyskerin and GAR1 levels were normalized to the Myc-NHP2 levels in the same lane. The values obtained for Myc-NHP2-WT were set to 1, and all the other values were calculated relative to it. (**C**) Immunoblot for NHP2 and (**D**) quantification of endo-NHP2 or Myc-NHP2^*^ in HT1080 cells transduced with EV or the indicated *MYC-NHP2* alleles before or after CRISPR/Cas9-mediated deletion of endo-*NHP2* using actin as loading control for *n* = 3 independent experiments, with average and SD. The signal for endo-NHP2 and Myc-NHP2^*^ was normalized to actin. The EV normalized value was set to 1 and all the other values were calculated relative to it. (**E**) Immunoblot for NHP2 and (**F**) quantification in HT1080 cells expressing the indicated *MYC-NHP2* alleles, with or without treatment with bortezomib for 6 h. Quantification as in (D) for *n* = 3 independent experiments, with average and SD. (**G**) Immunoblot for Dyskerin, NOP10 NHP2, and actin as loading control after depletion of Dyskerin with two different shRNA. (**H**) Quantification as in (G) for n= 3 independent experiments with average and SD. For each protein, the levels were first normalized to actin and then to the value obtained for the control (-).(**I**) Immunoblot of Dyskerin and endo-NHP2/Myc-NHP2^*^ 6 h after bortezomib treatment in HT1080 cells transduced with the indicated *MYC-NHP2* alleles before and after pull-down with anti-Dyskerin antibody. (**J**) Quantification for *n* = 3 independent experiments of the ratio of endo-NHP2 and Myc-NHP2^*^ versus total NHP2^*^ in each input and IP lane as shown in (G). Statistics: (B) and (H) ordinary one-way ANOVA for multiple comparisons for each protein, (D) unpaired *t*-test, (F) ratio paired *t*-test for each Myc-NHP2 variant, (J) two-way ANOVA for multiple comparisons for each Myc-NHP2^*^.

In parallel, the five initial models predicted by RoseTTAFold for either NHP2-A39T or NHP2-T44M (Supplementary Materials, Fig. S4D and H) were also used as starting points for five 1 μs-long MD simulations. Neither mutations affected the bulk of NHP2 structure; however, there were substantial differences in the MD simulations of the N-terminal, with several changes in the hydrogen-bonds network (Fig. 3, Supplementary Material, Figs S4 and S5, and Supplementary Material, Movies S6–S15). Indeed, NHP2-A39T was slightly more flexible than NHP2-WT, with the most frequent conformation, accounting for 53% of all the structures adopted during the simulation, showing an aberrant torsion of the amino acids 33–41 (Fig. 3B and F, Supplementary Material, Fig. S4E–G, and Supplementary Material, Movies S6–S10), while the positioning of the upstream N-terminal tail was similar to NHP2-WT (Fig. 3G). The second conformation, accounting for 14% of the adopted structures, showed instead the first 24 amino acids invading the interface between NHP2 and NOP10 and hTR (Fig. 3B and H and Supplementary Material, Fig. S4E), whereas the polypeptide chain folded as in NHP2-WT after residue P33 (Fig. 3F and Supplementary Material, Fig. S4F). For NHP2-T44M, instead, the MD simulation revealed a very broad cluster distribution, with the first two conformations representing together only 41% of the explored conformations (Fig. 4B and Supplementary Material, Movies S11–S15). This broad distribution suggests a much higher flexibility of the N-terminal in this mutant, probably caused by the loss of the interaction between T44 and S40 (Supplementary Material, Fig. S5). Notably, in this latter mutant, also residue R41, reported as mutated to histidine in another TBD patient (29), loses its recurrent interaction with E16. Furthermore, both the most explored conformations for T44M caused the insertion of the N-terminal 24 amino acids along NHP2 and NOP10/hTR without affecting the Q25-R41 loop (Fig. 3I–K and Supplementary Material, Fig. S4I and J).

In conclusion, this *in silico* analysis predicts that neither mutations compromise the folding of NHP2 core. Instead, they alter the behavior of the flexible N-terminal region of NHP2 by affecting its hydrogen-bonds networks. Although this could potentially reduce the intrinsic stability of NHP2, MD simulation suggests instead that the torsion of residues 33–41 and/or the insertion of the N-terminal in the interface between NHP2 and NOP10 would reduce the affinity of both variants for NOP10.

### NHP2-A39T and NHP2-T44M are outcompeted and highly degraded in the presence of wild-type NHP2

To test this hypothesis, we performed co-immunoprecipita-tion (Co-IP) against Myc in HT1080 cells expressing either NHP2-WT, NHP2-A39T or NHP2-T44M after deletion of endo-*NHP2*. However, consistently with their ability to support cell survival (Supplementary Material, Fig. S2B) and the relatively late onset of TBD in the index patient, both variants interacted with NOP10, Dyskerin and GAR1 to similar, if not higher, levels than NHP2-WT (Fig. 4A and B), demonstrating that neither of the substitutions abolishes the possibility of NHP2 to be incorporated in the complex *per se*.

Nevertheless, we noticed that before deleting endogenous NHP2, the levels of NHP2-WT represented about 50% of the total NHP2 extracted from HT1080 cells, whereas NHP2-A39T and NHP2-T44M levels represented less than 1% and 10% of total NHP2, respectively (Fig. 4C and D), and NHP2-WT expression caused a reduction in the levels of endo-NHP2 of about 50%, while the two mutated variants did not affect the level of endo-NHP2 (Fig. 4C and D). Furthermore, deletion of endogenous *NHP2* with lentiviral CRISPR Cas9/sgRNA caused a significant increase in the levels of NHP2-A39T and NHP2-T44M, about 50- and 4-fold, respectively, whereas only slightly affecting NHP2-WT levels (Fig. 4C and D). Similarly, treatment with the proteasome inhibitor bortezomib increased the levels of NHP2-A39T and NHP2-T44M by about 20- and 3-fold, respectively, whereas it did not affect the levels of endo-NHP2 or NHP2-WT (Fig. 4E and F), suggesting that both NHP2-A39T and NHP2-T44M are subject to high proteasomal degradation in cells expressing wild-type NHP2, whereas they are stabilized when no wild-type NHP2 is present.

Because our *in silico* analysis predicted a reduction in the affinity of the two NHP2 variants for NOP10 and depletion of Dyskerin caused a reduction in NHP2 levels (Fig. 4G and H) (26), we investigated the possible correlation between NHP2-A39T and NHP2-T44M degradation in the presence of endo-NHP2 with the lack of incorporation in the H/ACA RNA binding complex. To this end, we stabilized Myc-NHP2 variants with bortezomib in cells expressing endo-NHP2 before performing Co-IP against endogenous Dyskerin. As expected, NHP2-WT was equally present in the cell extract and in the Co-IP fraction, representing about 70% of total NHP2 in both (Fig. 4I and J). On the contrary, the ratio of NHP2-A39T and NHP2-T44M in the total cell extract versus the Co-IP fraction decreased from about 50% to less than 15% and from about 60% to about 40%, respectively (Fig. 4I and J), demonstrating that both A39T and T44M substitutions reduce the binding affinity of NHP2 for NOP10/Dyskerin and that the lack of incorporation in the H/ACA RNA binding complex determines the rapid degradation of NHP2.

Importantly, similar competition between endo-NHP2 and exogenous NHP2 and the instability of NHP2-A39T and NHP2-T44M was also observed in U-2OS, HeLa cancer cells and in normal BJ fibroblasts (Supplementary Material, Figs S3D and S6A, B). NHP2-WT levels could range from about 6% of the endo-NHP2 in HeLa cells to 90% in U-2OS cells, whereas the levels of endo-NHP2 were almost unaffected by the expression of MYC-NHP2-WT in HeLa or U-2OS cells but were reduced below detection in BJ cells (Supplementary Material, Figs S3D and S6A, B). Although the reason for this variability in the different cell lines is still unknown, this indicates that NHP2 levels are highly regulated post-transcriptionally independently of the telomerase status.

### NHP2 N-terminal domain increases the binding affinity of NHP2 for NOP10

**Figure 5:**
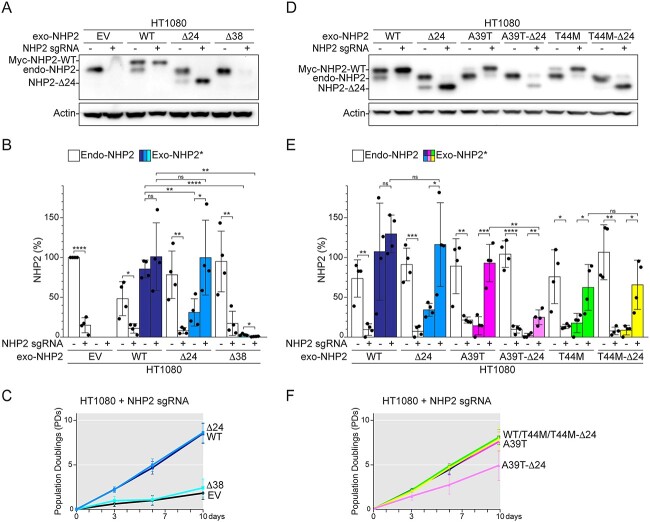
The N-terminal region of NHP2 promotes the incorporation into the H/ACA RNA binding complex and protein stability. Immunoblot (**A**) and quantification (**B**) for NHP2 expression in HT1080 cells transduced the empty vector (EV) or *MYC*-tagged *NHP2-WT*, *NHP2-Δ24* or *NHP2-Δ38*, before and after CRISPR/Cas9-mediated deletion of endo-*NHP2*. Actin was used as loading control. Graph shows *n* = 4 independent experiments with average and SD. (**C**) Survival curve for HT1080 cells treated as in (A). Day 0 is set 3 days after endo *NHP2* deletion. *n* = 4 independent experiments. Immunoblot for NHP2 (**D**), quantification of NHP2 over *n* = 3 independent experiment (**E**) and survival curve over *n* = 3 independent experiments (**F**) of HT1080 cells transduced with the indicated *MYC-NHP2* alleles before and after CRISPR/Cas9-mediated deletion of endo-*NHP2* as described in (A–C). The three experiments shown in (E) and (F) were performed together with three over four experiments shown in (B) and (C). Statistical analysis by unpaired *t*-test.

Because both variants were predicted to affect the positioning/folding of the NHP2 N-terminal, we investigated the role of the N-terminal in the formation of the H/ACA RNA binding complex by expressing untagged NHP2 mutants deleted of amino acids 2–24 (NHP2-Δ24) or amino acids 2–38 (NHP2-Δ38) before or after deletion of endo-NHP2 (Fig. 5A). NHP2-Δ24 was expressed significantly less than full-length NHP2 in the presence of endo-NHP2, but reached similar levels as MYC-NHP2-WT after deletion of endo-NHP2 (Fig. 5A and B). On the contrary, NHP2-Δ38 remained below detection in either the presence or absence of endo-NHP2, and this was not due to reduced transcription (Fig. 5A and B and Supplementary Material, Fig. S6C and Supplementary Material, Table S5). Consistently, NHP2-Δ24, but not NHP2-Δ38, complemented the lethality due to endo-NHP2 deletion (Fig. 5C). These data suggest that the correct folding of the first 23 amino acids of NHP2 is not strictly required for intrinsic protein stability, but to increase NHP2 affinity for NOP10/Dyskerin. On the contrary, residues 25–40 are necessary for NHP2 stability, intrinsically and/or by allowing the incorporation of NHP2 into the H/ACA RNA binding complex.

Importantly, either NHP2-A39T or NHP2-T44M variants were stabilized by the deletion of amino acids 2–24 (NHP2-A39T-Δ24 and NHP2-T44M-Δ24, respectively) (Fig. 5D and E), suggesting that the primary stability defect associated with both mutations is unlikely just due to the insertion of the N-terminal in the interface between NHP2 and NOP10, but rather to the general mis-positioning of the N-terminal. Consistently, Δ24 and T44M mutations are epistatic, suggesting that T44M substitution prevents the 23 N-terminal amino acids from promoting the H/ACA RNA binding complex assembly. On the contrary, Δ24 mutation was additive to A39T mutation both in terms of protein stability and cell survival (Fig. 5D–F), suggesting that the role of the first 23 amino acids in the formation of the H/ACA RNA binding complex is absolutely necessary in the presence of the A39T mutation.

## Discussion

Germline predisposition for bone marrow failure and hematological disorders, including malignancies, is a major clinical challenge, and numerous novel mutations have been identified during the last decade, including many associated with telomere biology (6–9). Identifying such conditions is of primary importance both for the patient and the rest of the family, preventing underdiagnoses and mistreatment, especially considering that some mutations can be asymptomatic in early years and lead to the late onset of TBDs even in heterozygosis (39–41). Furthermore, because for many of these disorders, including TBDs, there is currently no other treatment than allogeneic hematopoietic stem cell transplantation (42), the delay in the identification of the causative germline mutation can fatally affect the choice of donor and the conditioning regimen, as unfortunately it was in this case.

We herein report two novel variants in the *NHP2* gene (OMIM #613987), *NHP2-A39T* and *NHP2-T44M*, identified in a patient diagnosed with pancytopenia and signs of early aging. Both variants affect hTR stability and telomerase activity, validating them as pathogenic for TBDs. However, and consistent with the late onset of TBD, neither of the variants compromises the essential functions of NHP2. A specific impairment in binding hTR versus other H/ACA RNAs could explain the different effect on telomerase activity versus cell survival for both mutations. Another possibility is that lower NHP2 levels, as observed for both mutants even in the absence of wild-type NHP2, are sufficient to perform NHP2’s primary functions but are not enough for the secondary functions such as telomerase formation. Because NHP2-A39T is highly unstable in the presence of wild-type NHP2 while NHP2-T44M is still, at least partially, incorporated in the complex in the presence of wild-type NHP2, the *NHP2-A39T* could be reasonably considered recessive, whereas *NHP2-T44M* could be co-dominant. Although further analysis on individuals carrying *NHP2-T44M* in heterozygosity will be needed to confirm this hypothesis, this proposed analysis would be in agreement with the previous identification of monoallelic *NHP2* variants in adult patients diagnosed with AA and/or IPF (29).

Differently from what was previously shown for NHP2 depletion (26,27), neither NHP2 variants affects sensitivity to DNA damaging agents; therefore, further studies will be required to elucidate if and how NHP2 mutations could have contributed to malignancy. Indeed, while MDS may have occurred due to clonal hematopoiesis caused by telomere shortening, as previously reported (9), the etiology of the gastric cancer remains unknown, although it reminds the rare occurrence of solid cancers detected in male patients with Dyskerin mutations (9).

Importantly, our data present, for the first time, the relevance of the N-terminal region (residues 1–41) of NHP2 in promoting H/ACA RNA binding complex formation and NHP2 stability. Residues 2–24 are the most divergent between species and even absent in the archaea paralogue RL7. Furthermore, they have never been resolved by X-ray crystallography, cryo-EM (4) or NMR spectroscopy (43) due to their high disorder/flexibility and their tendency to dimerize in solution. Residues 25–41 are instead highly conserved and, although flexible, form a stable loop on the top of NHP2’s core, with multiple contact points with NOP10 (4). Here, we show that the first 23 amino acids of NHP2 promote the efficient incorporation of NHP2 into the H/ACA RNA binding complex, whereas the loop 25–38 is absolutely required for NHP2 stability. How this loop promotes NHP2 stability is still undetermined. It could have an intrinsic role in forming the NHP2 core, for example by sequestering the solvent, or it could have an indirect role by forming the interface with NOP10. Because, as we showed here, unbound NHP2 is massively degraded, we cannot discriminate between these two possibilities.

As for the first 23 amino acids, it is possible that they actively promote transient interactions with NOP10 and/or Dyskerin. Indeed, also Dyskerin has several unresolved regions that are predicted to be highly flexible and could mediate such interaction. Another non-mutually exclusive possibility is that the correct binding of this flexible N-terminal tail to the external surface of NHP2 stabilizes the folding of the 25–38 loop, therefore promoting the formation of the interface with NOP10. Because the addition of a Myc-tag before the N-terminal does not compromise NHP2’s stability and/or functions, we do favor the second hypothesis, but further studies will be required to characterize the dynamic mechanism underlying the interaction of NHP2 with NOP10/Dyskerin.

In this context, the telomerase-deficient phenotype observed in these new NHP2 variants could be explained by three non-mutually exclusive hypotheses: defective binding to hTR (i), low levels of active H/ACA RNA binding complex due to reduced intrinsic stability of NHP2 (ii) and/or defective incorporation of NHP2 into the complex (iii). In fact, both mutants are predicted to have reduced interaction with hTR but are also less expressed than wild-type NHP2. Furthermore, as discussed before, it is not possible to discriminate between intrinsic instability due to misfolding and proteasome-dependent degradation of all the NHP2 not incorporated into the H/ACA RNA binding complex. However, based on our *in silico* and genetic analysis, we would like to propose that the main effect of the T44M mutation is to increase the flexibility of the N-terminal aberrantly. This increased flexibility would then compromise the correct positioning of the N-terminal and its function in the formation of the H/ACA RNA binding complex. Furthermore, mispositioning of the N-terminal could also directly reduce the ability of NHP2 to bind hTR. The effect of A39T substitution seems instead to be more deleterious since the torsion of the P33-R41 loops could severely affect the interface between NHP2 and NOP10, unless the correct positioning of the first 23 amino acids compensates for it. This would explain why the removal of the N-terminal tail in the NHP2-A39T variant exacerbate NHP2 instability.

The lack of incorporation of NHP2 in the H/ACA RNA binding complex promotes dramatic NHP2 degradation. Tight regulation of NHP2 levels has been previously shown to be associated with ALT (27) cells. Here, we demonstrate that it is also relevant in untransformed fibroblasts and telomerase-positive cancer cell lines. Because NHP2 has many potential ubiquitination sites (15 out of 153 amino acids), one intriguing possibility is that, once bound to NOP10, these lysines would be hidden, whereas on free NHP2 they would be accessible to ubiquitination and signal NHP2 to degradation. However, further studies will be required to investigate if and why free NHP2 could affect cell fitness regardless of the telomerase status.

## Materials and Methods

### Telomere length analysis

Relative telomere length (RTL) was determined by quantitative PCR on 17.5 ng DNA extracted from peripheral blood, as described in (44), using single copy gene HBG as control and CFX96 instrument (BioRad, Hercules, CA). A standard curve of the reference cell line DNA is included in every run to monitor PCR efficiency,

### TruSight myeloid panel sequencing

Sequencing libraries were prepared from 50 ng of genomic DNA using TruSight Myeloid Sequencing Panel (FC-130-1010; Illumina, San Diego, CA) according to the manufacturer’s instructions. Ingenuity Variant Analysis (Qiagen) was used to detect SNVs and small indels with detection thresholds of 500× coverage, variant allele frequency of 5% and minimum 50 reads for variant. No variants met the conditions to be included in downstream analysis.

### Whole-genome sequencing

Sequencing libraries were prepared from 1 μg DNA using the TruSeq PCRfree DNA sample preparation kit (Illumina) targeting an insert size of 350 bp. The library was sequenced with paired-end 150 bp reads on NovaSeq S4 flowcell, with v1 sequencing chemistry (Illumina). Raw sequence reads were aligned to the human reference GRCh37.75 using bwa 0.7.12 (https://github.com/lh3/bwa). The alignments were deduplicated (PicardMarkDuplicates), recalibrated and indel realigned using **G**enome **A**nalysis **T**ool**k**it, GATK v3.3.0 (https://github.com/broadinstitute/gatk-docs). Small nucleotide variants and indels were called using the GATK Haplotypecaller and annotated using snpEff v4.1 (http://pcingola.github.io/SnpEff/). Variant files and patient HPO term descriptions were uploaded to Moon (Diploid, Leuven, Belgium) for variant prioritization. Highlighted variants were inspected using Integrative Genomics Viewer (Broad Institute, Cambridge, MA) (45).

### Alignment and sequence analysis

Protein sequences were obtained from BLAST-Uniprot (https://www.uniprot.org) and aligned in SnapGene (GSL Biotech) using Clustal Omega or MUSCLE multiple sequence comparison. Alignments were colored with ClustalX coloring (properties + conservation) or by identity using *Homo sapiens* NHP2 as reference.

The functional effect of substitution was calculated by SIFT (https://sift.bii.a-star.edu.sg) (46), where both substitutions were predicted to ‘affect protein function’ with a score of 0.00, or PolyPhen-2 (http://genetics.bwh.harvard.edu/pph2/dokuwiki/start) (47), where they were predicted to be ‘Probably damaging’ with a score of 0.999.

### Cell lines and cell treatments

HeLa S3, HT1080, U-2OS, BJ, 293T and Phoenix AMPHO (ATCC, Rockville, MD) were cultured in Dulbecco’s modified Eagle medium (DMEM) (Corning) supplemented with 10% fetal bovine serum (FBS) (Gibco), l-glutamine (Gibco), non-essential amino acids (Gibco) and penicillin–streptomycin (Gibco) at 37°C and 5% CO_2_. Bortezomib (PS-341; Selleckchem) was dissolved in DMSO (Sigma) and added to the cells (final concentration of 100 nm) for 6 h before harvest.

### Plasmid constructs and viral gene delivery

*NHP2-WT* was cloned in pWZL-MYC hygromycin-resistant retroviral vector (GenScript). The mutations *G115A* (NHP2-A39T) or *C131T* (NHP2-T44M) were inserted by site-directed mutagenesis. The gRNA against endogenous *NHP2* (5′-CAAATGCATCAAGAAAGGTG-3′) was cloned in pLentiCRISPR v2 puromycin-resistant lentiviral vector. Depletion of Dyskerin was obtained using shRNA cloned in pLKO.1 vector (shRNA.1: TRCN0000010324, target sequence: 5′-CACTATACACCTCTTGCATGT-3′; shRNA.2: TRCN0000352996, target sequence 5′-TATGTTGACTACAGTGAGTCT-3′; Sigma). Phoenix AMPHO and 293T were transfected by CaPO_4_ precipitation with 20 μg of plasmid DNA for Retroviral and Lentiviral transduction, respectively. Viral supernatant, supplemented with 4 μg/ml polybrene (Merck Millipore), was filtered through a 0.45-μm filter and used for transduction of target cells two/three times per day over 2 days. Target cells were selected in 300 μg/ml Hygromycin B (CAS 31282-04-9; Sigma) or 300 nm Puromycin (CAS 58-58-2; Merck) for 3–6 days.

Deletions of amino acids 2–24 or 1–37 were performed by PCR with the following primers:

NHP2—Rev: 5′-CATGGTGGCTGATCCGGCCGGC-3′.

NHP2-Δ24—For: 5′-CAGGAGCTGCTGGTCAACCAG-3′.

NHP2-Δ38—For: 5′-GCTTCTCGCCGCCTCACGCGG-3′.

### Immunoblotting and co-immunoprecipitation

For immunoblotting, cells were trypsinized and lysed in 2× Laemmli buffer (100 mm Tris–HCl pH 6.8, 200 μm DTT, 3% SDS, 20% glycerol, 0.05% bromophenol blue) at 5 × 10^3^ cells/μl. The lysate was denatured for 10 min at 96°C, and genomic DNA was sheared with 29-gauge needle. Proteins were separated by SDS/PAGE and transferred to a nitrocellulose membrane. The membrane was blocked in 5% milk dissolved in PBS with 0.1% Tween-20 and incubated with primary antibodies for 2 h at RT. Secondary anti-mouse or anti-rabbit IgG HRP antibodies (GE Healthcare) were incubated for 1 h at RT. The signal was detected by ChemiDoc (BioRad, Hercules, CA) after adding Chemilimunescence western blotting reagents (GE Healthcare) and quantified using ImageJ (48). Primary antibodies: Myc-tag (9B11, mAb 2276; Cell Signaling), NHP2 (ab180498; Abcam), GAR1 (NBP2–31742; Novus Biologicals), NOP10 (ab134902; Abcam), Dyskerin (ab156877; Abcam) and B-actin (8H10D10, mAb 3700; Cell Signaling).

For co-immunoprecipitation, cells were harvested by trypsinization, washed twice in PBS and then lysed in lysis buffer [50 mm Tris–HCl, 200 mm NaCl, 0.05% IGEPAL, Complete protease inhibitor mix (Roche) and PhosSTOP phosphatase inhibitor mix (Roche)] (49). After sonication for 2 × 5 s (40 amp), the lysates were incubated on ice for 10 min and then centrifuged at 4°C at full speed for 10 s. Then, 50 μl of the supernatant was saved as input. The rest of the supernatant was pre-cleared with beads for 2 h at 4°C, and then incubated overnight at 4°C with 6 μl of antibody. Finally, 60 μl of beads was added for 3 more hours at 4°C before three washes with lysis buffer. Laemmli buffer (2×) was added and samples were boiled for 10 min before gel loading.

### RNA extraction and qRT–PCR

RNA was extracted from 10^6^ cells using RNeasy Mini Kit (Qiagen). RNA (500 ng) was reverse transcribed using First Strand cDNA Synthesis Kit (Thermo Scientific). SYBR Green PCR Master Mix (Applied Biosystems) was used for qPCRs. BioRad software was used to automatically calculate thresholds. Primers used in this study were as follows:

GAPDH-F: 5′-GTCTCCTCTGACTTCAACAGCG-3′ (OriGene).

GAPDH-R: 5′-ACCACCCTGTTGCTGTAGCCAA-3′ (OriGene).

hTR-F: 5′-AACCCTAACTGAGAAGGGCG-3′.

hTR-R: 5′-AGAATGAACGGTGGAAGGCG-3′.

NHP2-F: 5′-AGACACACTGCCCATTGAGG-3′.

NHP2-R: 5′-TCCAGGCACTCATCGTAAGC-3′.

hTR or NHP2 values were normalized on GAPDH mRNA.

### Telomerase activity assay

Samples were prepared according to (50). Real-time quantitative telomeric repeat amplification protocol (RQ-TRAP) was performed as described in (51) in 20 μl final volume by two-step qPCR for 35 cycles (95°C, 30 s, 60°C, 90 s), after incubating 200 ng of protein extract with 10 μl 2× SYBR green master mix, 0.2 μm ACX primer and 0.3 μm TS primer (52) at 25°C for 20 min. The negative controls were incubated before the RQ-TRAP assay for 15 min at 85°C for telomerase inactivation. The standard curve was prepared by dilutions of TSR8 template according to the manufacturer’s instructions (Merck Millipore). BioRad software was used to automatically calculate thresholds.

### Colony assays

HeLa and U-2OS cells were seeded in 6-well plates in triplicate or duplicate at 100 or 250 cells per well, respectively. After 24 h, cells were incubated with aphidicolin (CAS 38966-21-1; Sigma) (50–300 nm, whole growth), neocarzinostatin (CAS 9014-02-2; Sigma) (12.5, 25, 50 ng/ml, 1 h), cisplatin (CAS 15663-27-1; Sigma) (0.5–2 μm, 24 h) or their respective controls (154 mm NaCl or DMSO; Sigma). For γ-irradiation, cells were exposed to 1–3 Gy/min. Cells were incubated for 10–14 days with one change of medium at day 6. Plates were stained with 50% methanol/2% methylene blue and colonies were scored manually.

### Disorder prediction

The predicted disordered agreement for NHP2 was obtained by entering hNHP2 sequence into the D^2^P^2^ disorder predictor database (http://d2p2.pro/) (38).

### Model generation and molecular dynamics

Five structures of full-length NHP2-WT, NHP2-A39T and NHP2-T44M were modeled using RoseTTAFold (37) at the Robetta webserver (https://robetta.bakerlab.org/), with no template provided and standard parameters. MD simulations were performed using GROMACS 2020.3 software package (www.gromacs.org) and CHARMM36m force field (53). Each model was solvated in a cubic box using TIP3P water model with a minimum distance between protein and box edges of 1 nm. Counterions K^+^ and Cl^−^ were added at a concentration of 100 mm to neutralize the net charge. For each protein ensemble, five simulations of 1 μs were carried out for each protein system (for a total time of simulation of 5 μs for each protein variant) within NPT thermodynamic ensemble at 300 K and 1 atm using a time step of 2 fs. Cluster analysis was then performed on a skipped trajectory of 5000 frames for each system. The RMSD matrices were computed on backbone atoms and then processed using the Gromos (54) algorithm with a cutoff of 0.50 nm. Hydrogen bonds were analyzed using the GROMACS Hydrogen-bond tool, taking into account the entire trajectory of 50 000 frames. The hydrogen bonds retrieved for the N-terminal 40 aa were represented as a network of interactions using Cytoscape 3.9.1. The representations of protein structures were built with Pymol Molecular Graphics System, version 2.0 (Schrödinger, LLC).

### Statistical analysis

GraphPad Prism was used to perform statistical analysis on at least three separate experiments.

Significance was assessed by calculating the *P*-value using unpaired *t*-test, ordinary one-way ANOVA for multiple comparisons, two-way ANOVA for multiple comparisons or ratio paired *t*-test. *P*-Values ≤0.05 were considered statistically significant. ns, not significant, ^*^P ≤ 0.05, ^*^^*^P ≤ 0.01, ^*^^*^^*^P ≤ 0.001, ^*^^*^^*^^*^P ≤ 0.0001.

## Supplementary Material

HMG-2023-CE-00181Malinski_Supplementary_Data_proof_ddad114

Supplemental_Material_Table_S1_ddad114

Supplemental_Material_Table_S2_ddad114

Supplemental_Material_Table_S3_ddad114

Supplemental_Material_Table_S4_ddad114

Supplemental_Material_Table_S5_ddad114

Movie_S1_ddad114

Movie_S2_ddad114

Movie_S3_ddad114

Movie_S4_ddad114

Movie_S5_ddad114

Movie_S6_ddad114

Movie_S7_ddad114

Movie_S8_ddad114

Movie_S9_ddad114

Movie_S10_ddad114

Movie_S11_ddad114

Movie_S12_ddad114

Movie_S13_ddad114

Movie_S14_ddad114

Movie_S15_ddad114

## Data Availability

All data generated or analyzed during this study are included in this article. The movies of the molecular dynamics experiments and the raw data for hTR expression and TRAP assays are available as Supplemental Material. All the other raw data are available from the corresponding authors upon reasonable request.

## References

[1] de Lange, T. (2018) Shelterin-mediated telomere protection. Annu. Rev. Genet., 52, 223–247. 30208292 10.1146/annurev-genet-032918-021921

[2] Roake, C.M. and Artandi, S.E. (2020) Regulation of human telomerase in homeostasis and disease. Nat. Rev. Mol. Cell. Biol., 21, 384–397. 32242127 10.1038/s41580-020-0234-z PMC7377944

[3] Greider, C.W. and Blackburn, E.H. (1985) Identification of a specific telomere terminal transferase activity in Tetrahymena extracts. Cell, 43, 405–413. 3907856 10.1016/0092-8674(85)90170-9

[4] Ghanim, G.E., Fountain, A.J., van Roon, A.-M.M., Rangan, R., Das, R., Collins, K. and Nguyen, T.H.D. (2021) Structure of human telomerase holoenzyme with bound telomeric DNA. Nature, 593, 449–453. 33883742 10.1038/s41586-021-03415-4 PMC7610991

[5] Revy, P., Kannengiesser, C. and Bertuch, A.A. (2023) Genetics of human telomere biology disorders. Nat. Rev. Genet., 24, 86–108. 36151328 10.1038/s41576-022-00527-z

[6] Alter, B.P., Giri, N., Savage, S.A. and Rosenberg, P.S. (2018) Cancer in the National Cancer Institute inherited bone marrow failure syndrome cohort after fifteen years of follow-up. Haematologica, 103, 30–39. 29051281 10.3324/haematol.2017.178111 PMC5777188

[7] Alter, B.P., Giri, N., Savage, S.A. and Rosenberg, P.S. (2009) Cancer in dyskeratosis congenita. Blood, 113, 6549–6557. 19282459 10.1182/blood-2008-12-192880 PMC2710915

[8] Reilly, C.R., Myllymäki, M., Redd, R., Padmanaban, S., Karunakaran, D., Tesmer, V., Tsai, F.D., Gibson, C.J., Rana, H.Q., Zhong, L. et al. (2021) The clinical and functional effects of TERT variants in myelodysplastic syndrome. Blood, 138, 898–911. 34019641 10.1182/blood.2021011075 PMC8432045

[9] Schratz, K.E., Haley, L., Danoff, S.K., Blackford, A.L., DeZern, A.E., Gocke, C.D., Duffield, A.S. and Armanios, M. (2020) Cancer spectrum and outcomes in the Mendelian short telomere syndromes. Blood, 135, 1946–1956. 32076714 10.1182/blood.2019003264 PMC7256360

[10] Arias-Salgado, E.G., Galvez, E., Planas-Cerezales, L., Pintado-Berninches, L., Vallespin, E., Martinez, P., Carrillo, J., Iarriccio, L., Ruiz-Llobet, A., Catalá, A. et al. (2019) Genetic analyses of aplastic anemia and idiopathic pulmonary fibrosis patients with short telomeres, possible implication of DNA-repair genes. Orphanet J. Rare Dis., 14, 82. 30995915 10.1186/s13023-019-1046-0 PMC6471801

[11] Holohan, B., Wright, W.E. and Shay, J.W. (2014) Cell biology of disease: telomeropathies: an emerging spectrum disorder. J. Cell Biol., 205, 289–299. 24821837 10.1083/jcb.201401012 PMC4018777

[12] Darzacq, X., Kittur, N., Roy, S., Shav-Tal, Y., Singer, R.H. and Meier, U.T. (2006) Stepwise RNP assembly at the site of H/ACA RNA transcription in human cells. J. Cell Biol., 173, 207–218. 16618814 10.1083/jcb.200601105 PMC2063812

[13] Fu, D. and Collins, K. (2003) Distinct biogenesis pathways for human telomerase RNA and H/ACA small nucleolar RNAs. Mol. Cell, 11, 1361–1372. 12769858 10.1016/s1097-2765(03)00196-5

[14] Richard, P., Kiss, A.M., Darzacq, X. and Kiss, T. (2006) Cotranscriptional recognition of human intronic box H/ACA snoRNAs occurs in a splicing-independent manner. Mol. Cell. Biol., 26, 2540–2549. 16537900 10.1128/MCB.26.7.2540-2549.2006 PMC1430331

[15] Wang, C. and Meier, U.T. (2004) Architecture and assembly of mammalian H/ACA small nucleolar and telomerase ribonucleoproteins. EMBO J., 23, 1857–1867. 15044956 10.1038/sj.emboj.7600181 PMC394235

[16] Bellodi, C., McMahon, M., Contreras, A., Juliano, D., Kopmar, N., Nakamura, T., Maltby, D., Burlingame, A., Savage, S.A., Shimamura, A. et al. (2013) H/ACA small RNA dysfunctions in disease reveal key roles for noncoding RNA modifications in hematopoietic stem cell differentiation. Cell Rep., 3, 1493–1502. 23707062 10.1016/j.celrep.2013.04.030 PMC3857015

[17] Bortolin, M.L., Ganot, P. and Kiss, T. (1999) Elements essential for accumulation and function of small nucleolar RNAs directing site-specific pseudouridylation of ribosomal RNAs. EMBO J., 18, 457–469. 9889201 10.1093/emboj/18.2.457 PMC1171139

[18] Fayet-Lebaron, E., Atzorn, V., Henry, Y. and Kiss, T. (2009) 18S rRNA processing requires base pairings of snR30 H/ACA snoRNA to eukaryote-specific 18S sequences. EMBO J., 28, 1260–1270. 19322192 10.1038/emboj.2009.79 PMC2664660

[19] Henras, A., Henry, Y., Bousquet-Antonelli, C., Noaillac-Depeyre, J., Gélugne, J.P. and Caizergues-Ferrer, M. (1998) Nhp2p and Nop10p are essential for the function of H/ACA snoRNPs. EMBO J., 17, 7078–7090. 9843512 10.1093/emboj/17.23.7078 PMC1171055

[20] Kiss, T., Fayet-Lebaron, E. and Jády, B.E. (2010) Box H/ACA small ribonucleoproteins. Mol. Cell, 37, 597–606. 20227365 10.1016/j.molcel.2010.01.032

[21] Yu, Y.-T. and Meier, U.T. (2014) RNA-guided isomerization of uridine to pseudouridine—pseudouridylation. RNA Biol., 11, 1483–1494. 25590339 10.4161/15476286.2014.972855 PMC4615163

[22] Egan, E.D. and Collins, K. (2010) Specificity and stoichiometry of subunit interactions in the human telomerase holoenzyme assembled in vivo. Mol. Cell. Biol., 30, 2775–2786. 20351177 10.1128/MCB.00151-10 PMC2876521

[23] Fu, D. and Collins, K. (2007) Purification of human telomerase complexes identifies factors involved in telomerase biogenesis and telomere length regulation. Mol. Cell, 28, 773–785. 18082603 10.1016/j.molcel.2007.09.023 PMC2917595

[24] Jiang, J., Wang, Y., Sušac, L., Chan, H., Basu, R., Zhou, Z.H. and Feigon, J. (2018) Structure of telomerase with telomeric DNA. Cell, 173, 1179–1190.e13. 29775593 10.1016/j.cell.2018.04.038 PMC5995583

[25] Nguyen, T.H.D., Tam, J., Wu, R.A., Greber, B.J., Toso, D., Nogales, E. and Collins, K. (2018) Cryo-EM structure of substrate-bound human telomerase holoenzyme. Nature, 557, 190–195. 29695869 10.1038/s41586-018-0062-x PMC6223129

[26] Lin, P., Mobasher, M.E., Hakakian, Y., Kakarla, V., Naseem, A.F., Ziai, H. and Alawi, F. (2015) Differential requirements for H/ACA ribonucleoprotein components in cell proliferation and response to DNA damage. Histochem. Cell Biol., 144, 543–558. 26265134 10.1007/s00418-015-1359-6 PMC5662186

[27] Raghunandan, M., Geelen, D., Majerova, E. and Decottignies, A. (2021) NHP2 downregulation counteracts hTR-mediated activation of the DNA damage response at ALT telomeres. EMBO J., 40, e106336. 33595114 10.15252/embj.2020106336 PMC7957427

[28] Wang, X.-C., Yue, X., Zhang, R.-X., Liu, T.-Y., Pan, Z.-Z., Yang, M.-J., Lu, Z.-H., Wang, Z.-Y., Peng, J.-H., Le, L.-Y. et al. (2019) Genome-wide RNAi screening identifies RFC4 as a factor that mediates radioresistance in colorectal cancer by facilitating nonhomologous end joining repair. Clin. Cancer Res., 25, 4567–4579. 30979744 10.1158/1078-0432.CCR-18-3735

[29] Benyelles, M., O’Donohue, M.-F., Kermasson, L., Lainey, E., Borie, R., Lagresle-Peyrou, C., Nunes, H., Cazelles, C., Fourrage, C., Ollivier, E. et al. (2020) NHP2 deficiency impairs rRNA biogenesis and causes pulmonary fibrosis and Høyeraal-Hreidarsson syndrome. Hum. Mol. Genet., 29, 907–922. 31985013 10.1093/hmg/ddaa011

[30] Heiss, N.S., Knight, S.W., Vulliamy, T.J., Klauck, S.M., Wiemann, S., Mason, P.J., Poustka, A. and Dokal, I. (1998) X-linked dyskeratosis congenita is caused by mutations in a highly conserved gene with putative nucleolar functions. Nat. Genet., 19, 32–38. 9590285 10.1038/ng0598-32

[31] Vulliamy, T., Beswick, R., Kirwan, M., Marrone, A., Digweed, M., Walne, A. and Dokal, I. (2008) Mutations in the telomerase component NHP2 cause the premature ageing syndrome dyskeratosis congenita. PNAS, 105, 8073–8078. 18523010 10.1073/pnas.0800042105 PMC2430361

[32] Walne, A.J., Vulliamy, T., Marrone, A., Beswick, R., Kirwan, M., Masunari, Y., Al-Qurashi, F.-H., Aljurf, M. and Dokal, I. (2007) Genetic heterogeneity in autosomal recessive dyskeratosis congenita with one subtype due to mutations in the telomerase-associated protein NOP10. Hum. Mol. Genet., 16, 1619–1629. 17507419 10.1093/hmg/ddm111 PMC2882227

[33] Karczewski, K.J., Francioli, L.C., Tiao, G., Cummings, B.B., Alföldi, J., Wang, Q., Collins, R.L., Laricchia, K.M., Ganna, A., Birnbaum, D.P. et al. (2020) The mutational constraint spectrum quantified from variation in 141,456 humans. Nature, 581, 434–443. 32461654 10.1038/s41586-020-2308-7 PMC7334197

[34] Gong, Y., Liu, Y., Wang, T., Li, Z., Gao, L., Chen, H., Shu, Y., Li, Y., Xu, H., Zhou, Z. et al. (2021) Age-associated proteomic signatures and potential clinically actionable targets of colorectal cancer. Mol. Cell. Proteomics, 20, 100115. 34129943 10.1016/j.mcpro.2021.100115 PMC8441843

[35] Tang, S., Wu, W., Wan, H., Wu, X. and Chen, H. (2020) Knockdown of NHP2 inhibits hepatitis B virus X protein-induced hepatocarcinogenesis via repressing TERT expression and disrupting the stability of telomerase complex. Aging (Albany NY), 12, 19365–19374. 33044946 10.18632/aging.103810 PMC7732313

[36] Dilley, R.L. and Greenberg, R.A. (2015) ALTernative telomere maintenance and cancer. Trends Cancer, 1, 145–156. 26645051 10.1016/j.trecan.2015.07.007 PMC4669901

[37] Baek, M., DiMaio, F., Anishchenko, I., Dauparas, J., Ovchinnikov, S., Lee, G.R., Wang, J., Cong, Q., Kinch, L.N., Schaeffer, R.D. et al. (2021) Accurate prediction of protein structures and interactions using a three-track neural network. Science, 373, 871–876. 34282049 10.1126/science.abj8754 PMC7612213

[38] Oates, M.E., Romero, P., Ishida, T., Ghalwash, M., Mizianty, M.J., Xue, B., Dosztányi, Z., Uversky, V.N., Obradovic, Z., Kurgan, L. et al. (2013) D2P2: database of disordered protein predictions. Nucleic Acids Res., 41, D508–D516. 23203878 10.1093/nar/gks1226 PMC3531159

[39] Armanios, M., Chen, J.-L., Chang, Y.-P.C., Brodsky, R.A., Hawkins, A., Griffin, C.A., Eshleman, J.R., Cohen, A.R., Chakravarti, A., Hamosh, A. et al. (2005) Haploinsufficiency of telomerase reverse transcriptase leads to anticipation in autosomal dominant dyskeratosis congenita. PNAS, 102, 15960–15964. 16247010 10.1073/pnas.0508124102 PMC1276104

[40] Vulliamy, T., Marrone, A., Szydlo, R., Walne, A., Mason, P.J. and Dokal, I. (2004) Disease anticipation is associated with progressive telomere shortening in families with dyskeratosis congenita due to mutations in TERC. Nat. Genet., 36, 447–449. 15098033 10.1038/ng1346

[41] Baliakas, P., Tesi, B., Wartiovaara-Kautto, U., Stray-Pedersen, A., Friis, L.S., Dybedal, I., Hovland, R., Jahnukainen, K., Raaschou-Jensen, K., Ljungman, P. et al. (2019) Nordic guidelines for germline predisposition to myeloid neoplasms in adults: recommendations for genetic diagnosis. Clinical management and follow-up. HemaSphere, 3, e321. 31976490 10.1097/HS9.0000000000000321 PMC6924562

[42] Young, N.S. (2012) Bone marrow failure and the new telomere diseases: practice and research. Hematology, 17(Suppl 1), S18–S21. 22507770 10.1179/102453312X13336169155132

[43] Koo, B.-K., Park, C.-J., Fernandez, C.F., Chim, N., Ding, Y., Chanfreau, G. and Feigon, J. (2011) Structure of H/ACA RNP protein Nhp2p reveals cis/trans isomerization of a conserved proline at the RNA and Nop10 binding interface. J. Mol. Biol., 411, 927–942. 21708174 10.1016/j.jmb.2011.06.022 PMC3156286

[44] Cawthon, R.M. (2002) Telomere measurement by quantitative PCR. Nucleic Acids Res., 30, e47. 12000852 10.1093/nar/30.10.e47 PMC115301

[45] Robinson, J.T., Thorvaldsdóttir, H., Winckler, W., Guttman, M., Lander, E.S., Getz, G. and Mesirov, J.P. (2011) Integrative genomics viewer. Nat. Biotechnol., 29, 24–26. 21221095 10.1038/nbt.1754 PMC3346182

[46] Ng, P.C. and Henikoff, S. (2001) Predicting deleterious amino acid substitutions. Genome Res., 11, 863–874. 11337480 10.1101/gr.176601 PMC311071

[47] Adzhubei, I.A., Schmidt, S., Peshkin, L., Ramensky, V.E., Gerasimova, A., Bork, P., Kondrashov, A.S. and Sunyaev, S.R. (2010) A method and server for predicting damaging missense mutations. Nat. Methods, 7, 248–249. 20354512 10.1038/nmeth0410-248 PMC2855889

[48] Schindelin, J., Arganda-Carreras, I., Frise, E., Kaynig, V., Longair, M., Pietzsch, T., Preibisch, S., Rueden, C., Saalfeld, S., Schmid, B. et al. (2012) Fiji: an open-source platform for biological-image analysis. Nat. Methods, 9, 676–682. 22743772 10.1038/nmeth.2019 PMC3855844

[49] Hoareau-Aveilla, C., Bonoli, M., Caizergues-Ferrer, M. and Henry, Y. (2006) hNaf1 is required for accumulation of human box H/ACA snoRNPs, scaRNPs, and telomerase. RNA, 12, 832–840. 16601202 10.1261/rna.2344106 PMC1440901

[50] Kim, N.W., Piatyszek, M.A., Prowse, K.R., Harley, C.B., West, M.D., Ho, P.L., Coviello, G.M., Wright, W.E., Weinrich, S.L. and Shay, J.W. (1994) Specific association of human telomerase activity with immortal cells and cancer. Science, 266, 2011–2015. 7605428 10.1126/science.7605428

[51] Wege, H., Chui, M.S., Le, H.T., Tran, J.M. and Zern, M.A. (2003) SYBR green real-time telomeric repeat amplification protocol for the rapid quantification of telomerase activity. Nucleic Acids Res., 31, e3. 12527792 10.1093/nar/gng003 PMC140528

[52] Kim, N.W. and Wu, F. (1997) Advances in quantification and characterization of telomerase activity by the telomeric repeat amplification protocol (TRAP). Nucleic Acids Res., 25, 2595–2597. 9185569 10.1093/nar/25.13.2595 PMC146790

[53] Huang, J., Rauscher, S., Nawrocki, G., Ran, T., Feig, M., de Groot, B.L., Grubmüller, H. and MacKerell, A.D. (2017) CHARMM36m: an improved force field for folded and intrinsically disordered proteins. Nat. Methods, 14, 71–73. 27819658 10.1038/nmeth.4067 PMC5199616

[54] Daura, X., Gademann, K., Jaun, B., Seebach, D., van Gunsteren, W.F. and Mark, A.E. (1999) Peptide folding: when simulation meets experiment. Angew. Chem. Int. Ed., 38, 236–240.

