# Supplementary material for: Novel pathological variants of NHP2 affect N-terminal domain flexibility, protein stability, H/ACA Ribonucleoprotein (RNP) complex formation and telomerase activity
Source: Hum Mol Genet. 2023 Jul 13;32(19):2901–12. doi: 10.1093/hmg/ddad114 (PMC10508036; doi:10.1093/hmg/ddad114)
Supplement: HMG-2023-CE-00181Malinski_Supplementary_Data_proof_ddad114 [file hmg-2023-ce-00181malinski_supplementary_data_proof_ddad114.docx]

**Supplementary Data.**

**Novel pathological variants of NHP2 affect N-terminal domain flexibility, protein stability, H/ACA RNP complex formation and telomerase activity**

Bartosz Malinski et al.

**
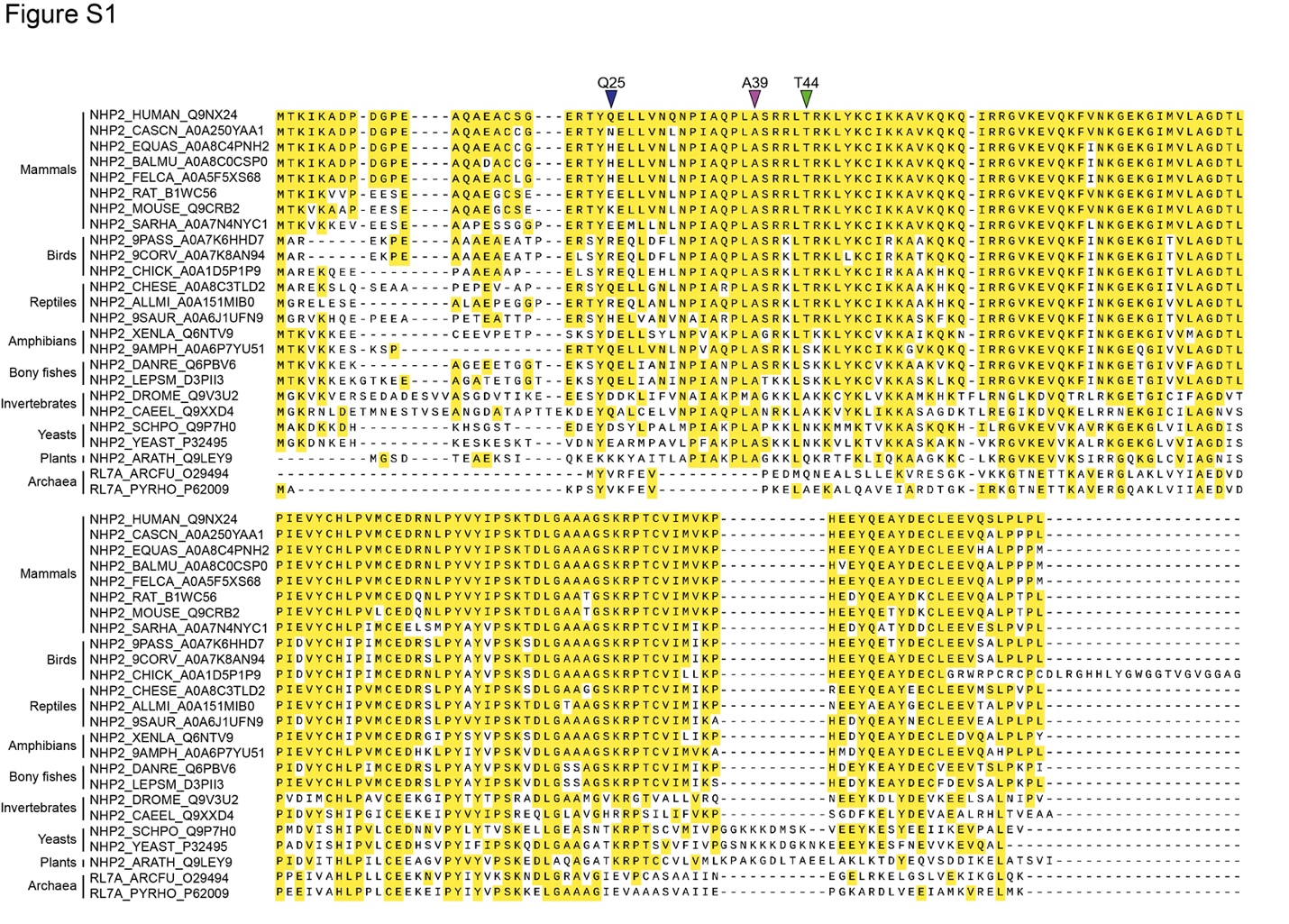
Supplementary Figure 1**

**Supplementary Figure 1.** MUSCLE Alignment of NHP2 isoforms from selected eukaryotes and RL7A from selected archaea, as indicated. In yellow are highlighted the residues identical to human NHP2. Arrows indicate Q25, A39 and T44, respectively.

**Supplementary Figure 2**

**
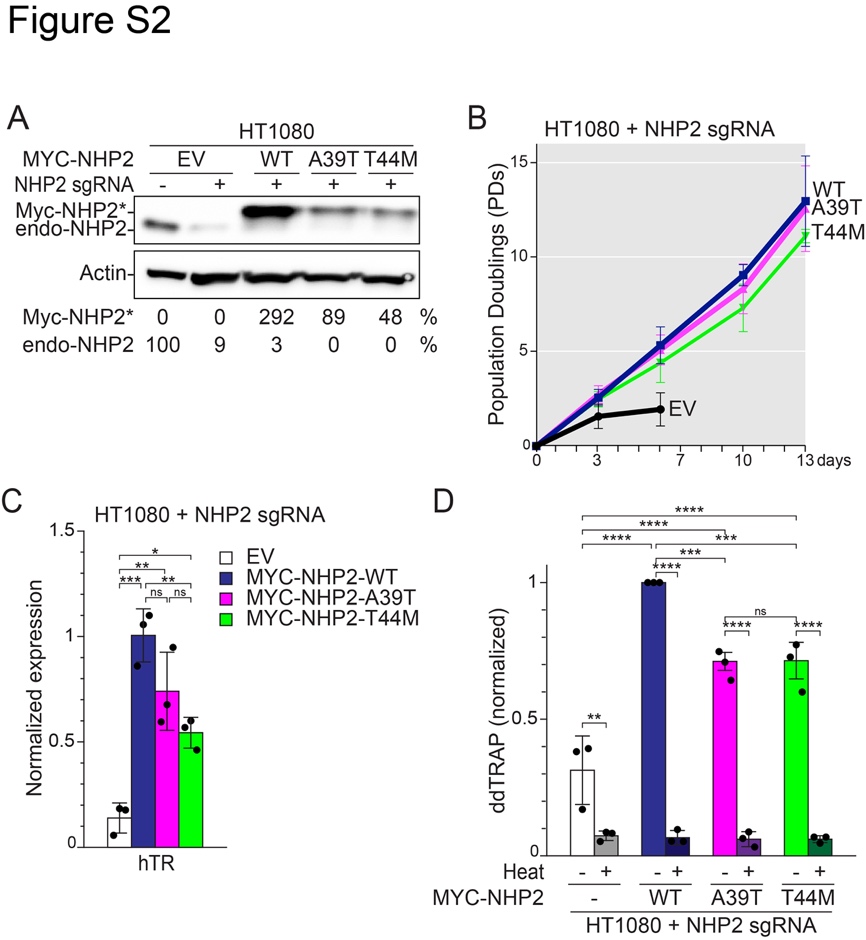
**
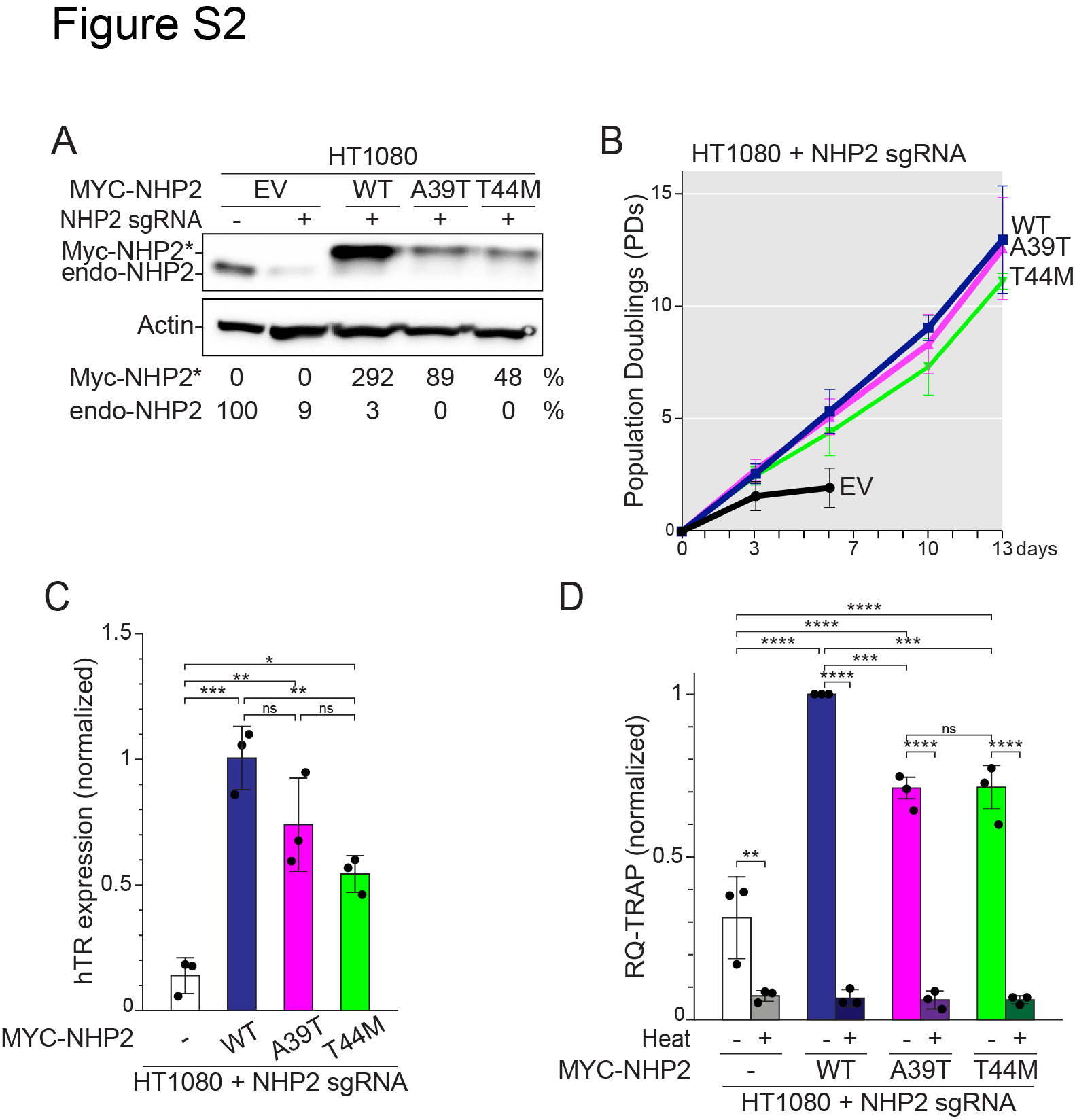


**Supplementary Figure 2.** Reduced telomerase activity in HT1080 cells expressing NHP2-A39T or NHP2-T44M. (**A**) Immunoblot for NHP2 and (**B**) survival curve in HT1080 cells transduced with the empty vector (EV) or *MYC*-tagged *NHP2-WT*, *NHP2-A39T* or *NHP2-T44M*, before and after deletion of endo-*NHP2*. The signal for Myc-NHP2 (Myc-NHP2*) or endogenous NHP2 (endo-NHP2) was normalized to the actin loading control and then to the value obtained for endo-NHP2 in untreated conditions. Day 0 is set 4 days after endo *NHP2* deletion. (**C**) hTR RNA and (**D**) Telomerase activity for n=3 independent experiments, with average and SD. Statistics: Ordinary one-way Anova for Multiple comparisons.

**Supplementary Figure 3**

**
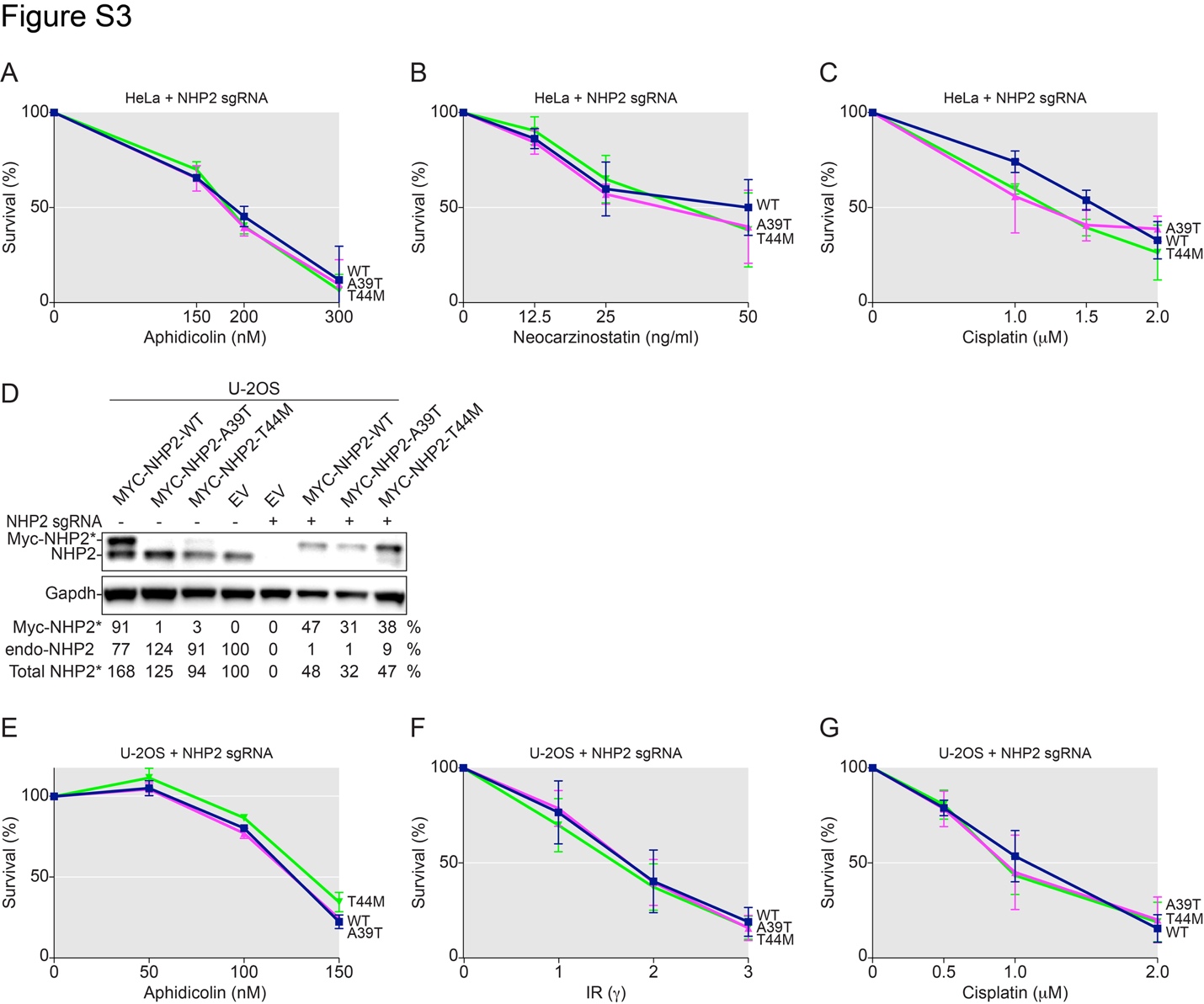
**

**Supplementary Figure 3.** Colony formation assay of HeLa cells transduced with *MYC*-tagged *NHP2-WT*, *NHP2-A39T* or *NHP2-T44M* after deletion of endo-*NHP2* and treatment with (**A**) Aphidicolin, (**B**) Neocarzinostatin, or (**C**) Cisplatin. (**D**) Immunoblot and quantification on loading control of Myc-NHP2* and endo-NHP2 in U-2OS cancer cells transduced with the empty vector or the indicated *MYC-NHP2* alleles before or after deletion of endo-*NHP2.* Colony assay of U-2OS cells transduced *MYC*-tagged *NHP2-WT*, *NHP2-A39T* or *NHP2-T44M* after deletion of endo-*NHP2* and treatment with (**E**) Aphidicolin, (**F**) γ-irradiation, or (**G**) Cisplatin. (**A-C**) and (**F-G**) Results from n=3 independent experiments, with SD. (**E**) Results from n=2 independent experiments with SEM.

**Supplementary Figure 4**


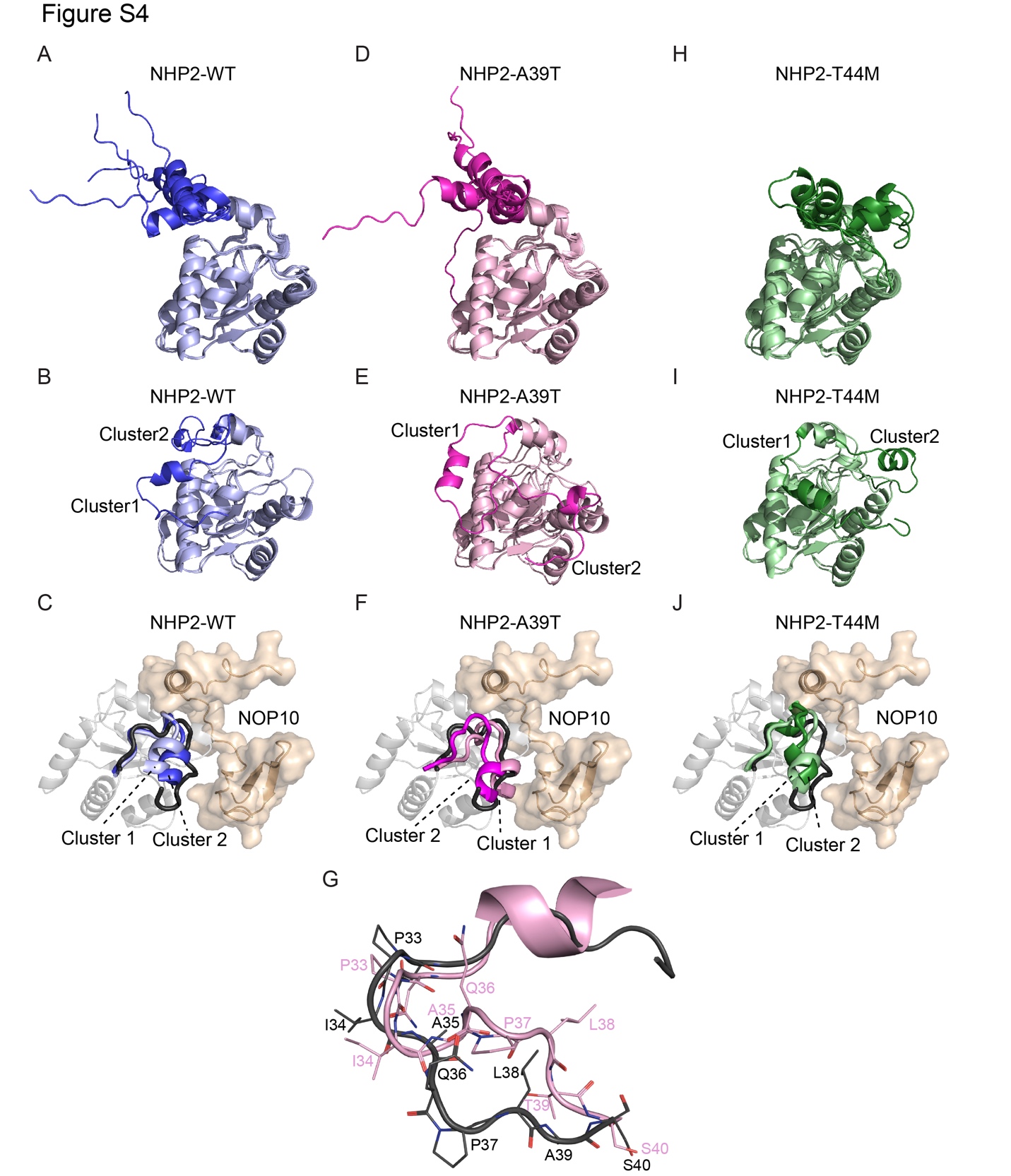


**Supplementary Figure 4.** Cartoon representation of (**A**) superposed 5 RoseTTAFold structure predictions and (**B**) centroids of the two more represented structural clusters in the trajectories of molecular dynamics (MD) simulation for wild-type NHP2. (**C**) centroids for wild-type NHP2 as in (**B**) superposed to the wild-type NHP2 (light and dark grey) and Nop10 (tan cartoon and surface) (PDB ID: 7bgb). Cartoon representation of (**D**) superposed 5 RoseTTAFold structure predictions and (**E**) centroids of the two more represented structural clusters in the trajectories of MD simulation for NHP2-A39T. (**F**) centroids for NHP2-A39T as in (**E**) superposed to the wild-type NHP2 (light and dark grey) and Nop10 (tan cartoon and surface) (PDB ID: 7bgb). (**G**) Detail of the altered structure of the loop comprised between aa 25 and aa 40, represented as a cartoon, from the centroid of the most represented structural cluster (cluster 1) in the trajectories of MD simulation for NHP2-A39T (light magenta), superimposed to the wild-type NHP2 corresponding loop (dark grey) (PDB ID: 7bgb). The residues from P33 to S40 are highlighted and represented as lines with red for oxygen and blue for nitrogen atoms. Cartoon representation of (**H**) superposed 5 RoseTTAFold structure predictions and (**I**) centroids of the two more represented structural clusters in the trajectories of MD simulation for NHP2-T44M. (**J**) centroids for NHP2-T44M as in (**I**) superposed to the wild-type NHP2 (light and dark grey) and Nop10 (tan cartoon and surface) (PDB ID: 7bgb).

**Supplementary Figure 5
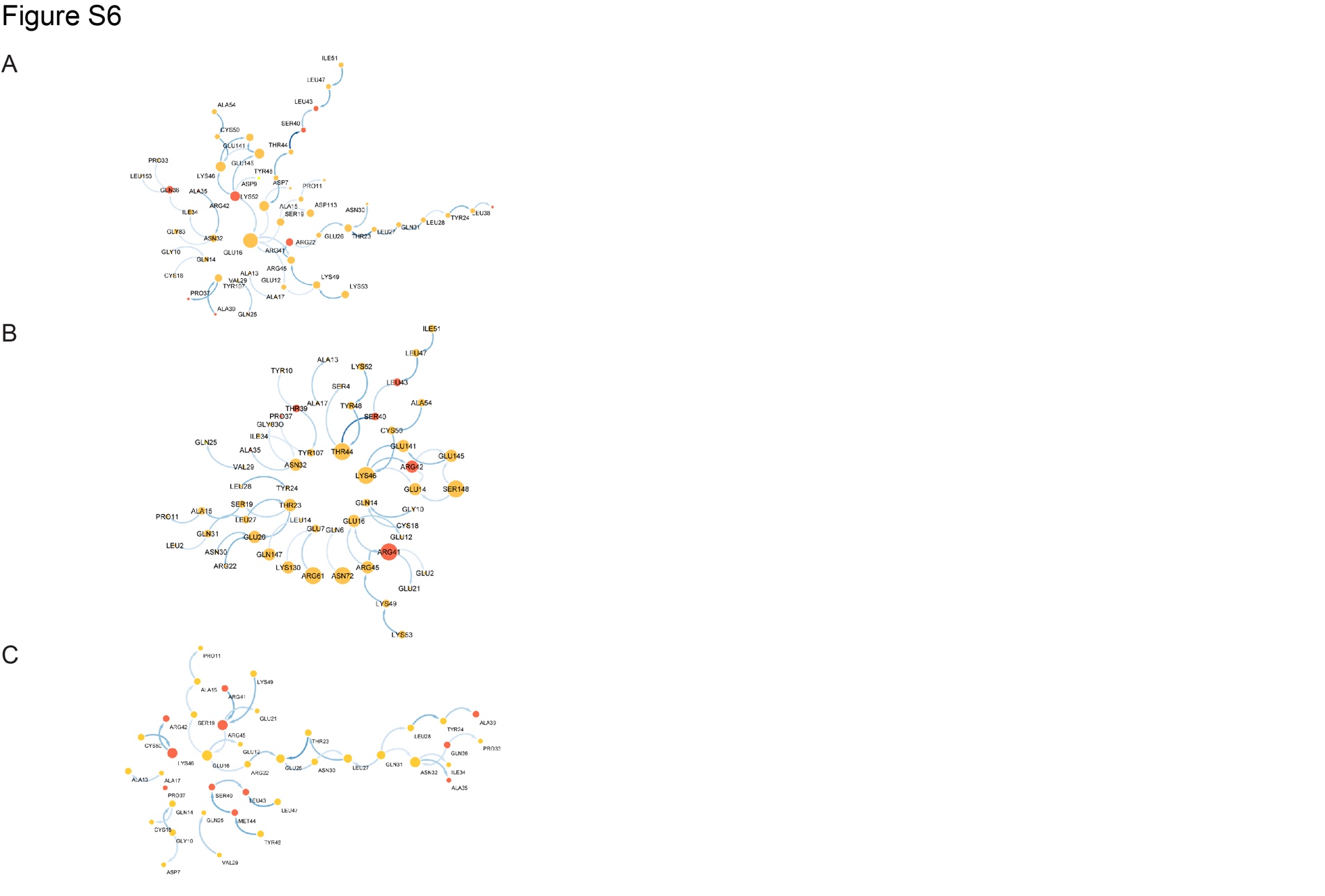
**

**Supplementary Figure 5.** Wheel visualization of the hydrogen-bond interactions in (**A**) wild-type NHP2, (**B**) NHP2-A39T, and (**C**) NHP2-T44M, as analyzed by GROMACS. The node size of each residue is proportional to the number of hydrogen-bonds detected in all 5 molecular dynamics simulation. The edge color represents the persistence of that hydrogen-bond, with light blue indicating low persistence, and dark blue high persistence. In red, are the residues surrounding A39 from A35 to T44. **Supplementary Figure 6**

**
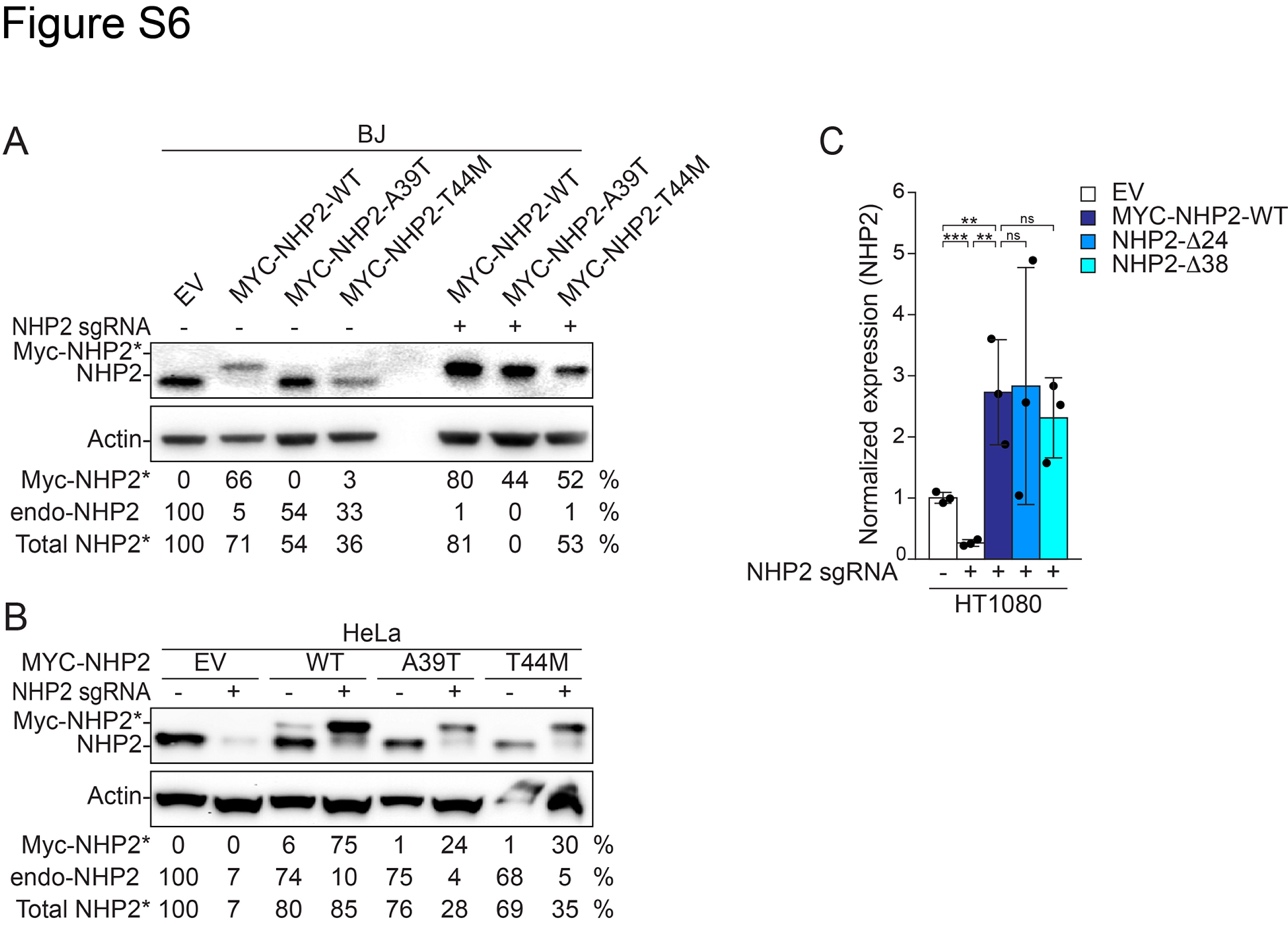
**

**Supplementary Figure 6.** Immunoblot and quantification on loading control of Myc-NHP2*, endo-NHP2 and total NHP2* in (**A**) BJ fibroblasts and (**B**) HeLa cancer cells transduced with the empty vector or the indicated *MYC-NHP2* alleles before or after deletion of endo-*NHP2*. Experiment with BJ fibroblast was done only once. (C) qPCR analysis of NHP2 expression in HT1080 cells transduced with the empty vector control (EV), *MYC-NHP2-WT*, *NHP2-Δ24* or *NHP2-Δ38*.
